# Hutchinson’s sign complicated by bilateral multiple cranial neuropathies and cerebral infarctions: a case report

**DOI:** 10.3389/fmed.2026.1745425

**Published:** 2026-03-10

**Authors:** Zhongzheng Chang, Ye Zhang, Jianwen Li, Cheng Zhang, Xiaolei Zheng, Shunliang Xu, Hui Yang, Zhaohong Xie

**Affiliations:** 1 Department of Neurology, The Second Qilu Hospital of Shandong University, Jinan, China; 2 Cheeloo College of Medicine of Shandong University, Jinan, China

**Keywords:** cerebral infarction, herpes zoster ophthalmicus, Hutchinson’s sign, multiple cranial neuropathies, varicella-zoster virus

## Abstract

Varicella-zoster virus (VZV) infection is associated with serious complications, including cranial vasculopathy and herpes zoster ophthalmicus (HZO). Hutchinson’s sign, characterized by VZV lesions on the tip, dorsum, and root of the nose, serves as a strong predictor of ipsilateral ocular complications in HZO. Here we describe a 61-year-old male who developed multiple cranial neuropathies and acute cerebral infarction secondary to VZV infection. His clinical manifestations began with Hutchinson’s sign, progressed to HZO, and subsequently led to acute cerebral infarction and bilateral ophthalmoplegia. The presence of Hutchinson’s sign strongly correlates with the development of HZO-related ocular complications. This striking bilateral cranial nerve involvement and cerebral infarctions suggest a potential mechanism of widespread viral dissemination within the central nervous system.

## Introduction

Varicella-zoster virus (VZV) infection can lead to a spectrum of complications, ranging from local sequelae such as herpes zoster ophthalmicus (HZO), iritis, and postherpetic neuralgia, to serious central nervous system (CNS) involvement; the latter includes vasculopathy, stroke, meningitis, meningoencephalitis, and self-limiting cerebellar ataxia (1). Hutchinson’s sign refers to cutaneous lesions on the tip, dorsum, and root of the nose caused by VZV infection. It is considered a strong predictor of the ipsilateral ocular complications of HZO (2). Anterior choroidal artery (AChA) infarction predominantly affects the posterior limb of the internal capsule and the globus pallidus (3). In this report, we present a rare case of Hutchinson’s sign complicated by bilateral multiple cranial neuropathies and AChA territory infarction, including the uncommon locations of the cerebral peduncle and parahippocampal gyrus. This striking bilateral cranial nerve involvement suggests a potential mechanism of widespread viral dissemination within the CNS. This extension of the AChA infarct beyond its classic territory illustrates the aggressive and diffuse nature of VZV-induced vasculopathy, which can affect the entire course of perforating arteries. We therefore emphasize the mechanisms underlying VZV-induced bilateral cranial nerve involvement and cerebral vasculopathy.

## Case presentation

**Figure 1:**
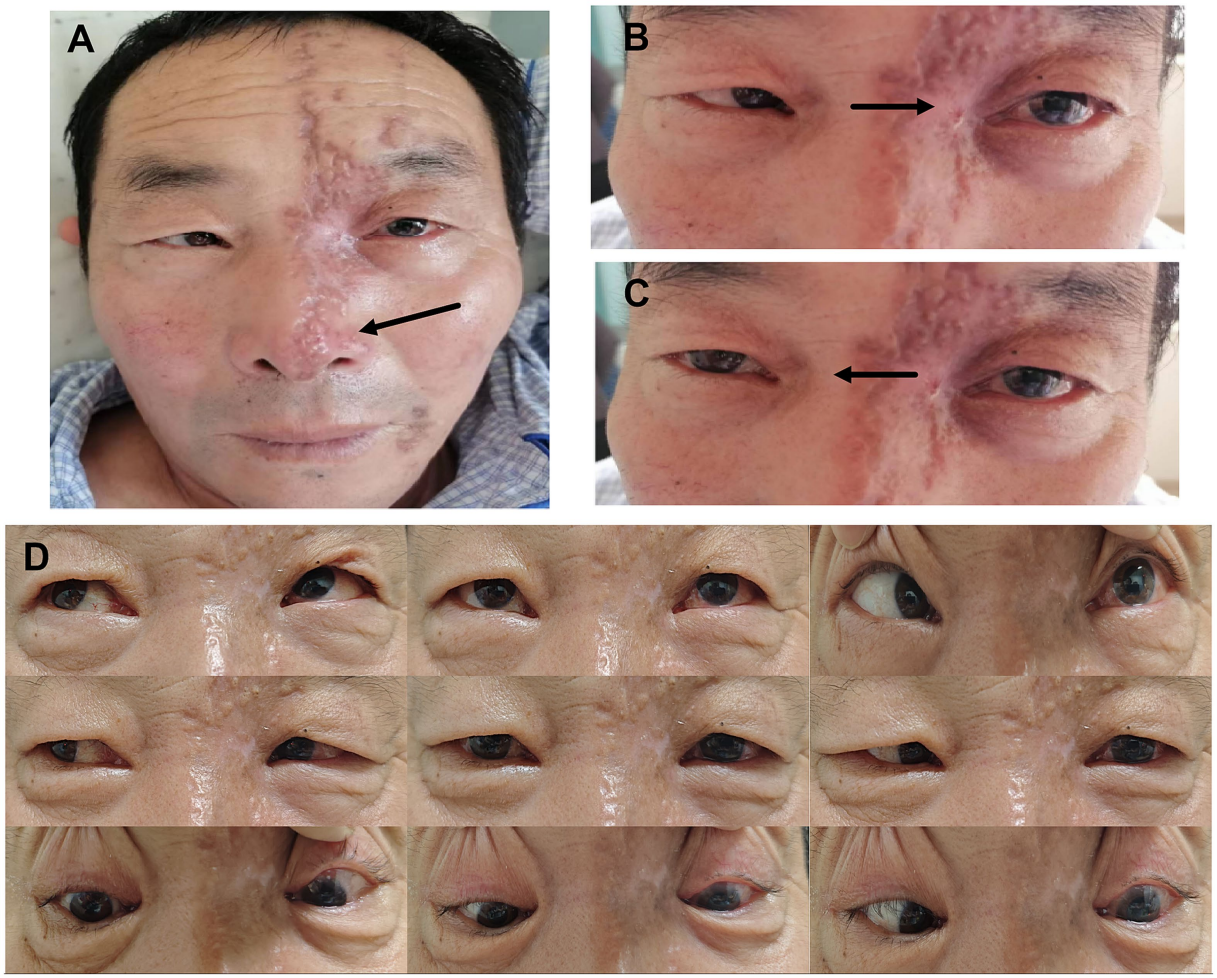
Patient’s facial and ocular motility images. **(A)** Hutchinson’s sign in herpes zoster ophthalmicus. **(B,C)** Left complete external ophthalmoplegia, and right eye abduction limitation. **(D)** Follow-up evaluation, nine cardinal directions of gaze, demonstrating impaired left eye abduction.

**Figure 2:**
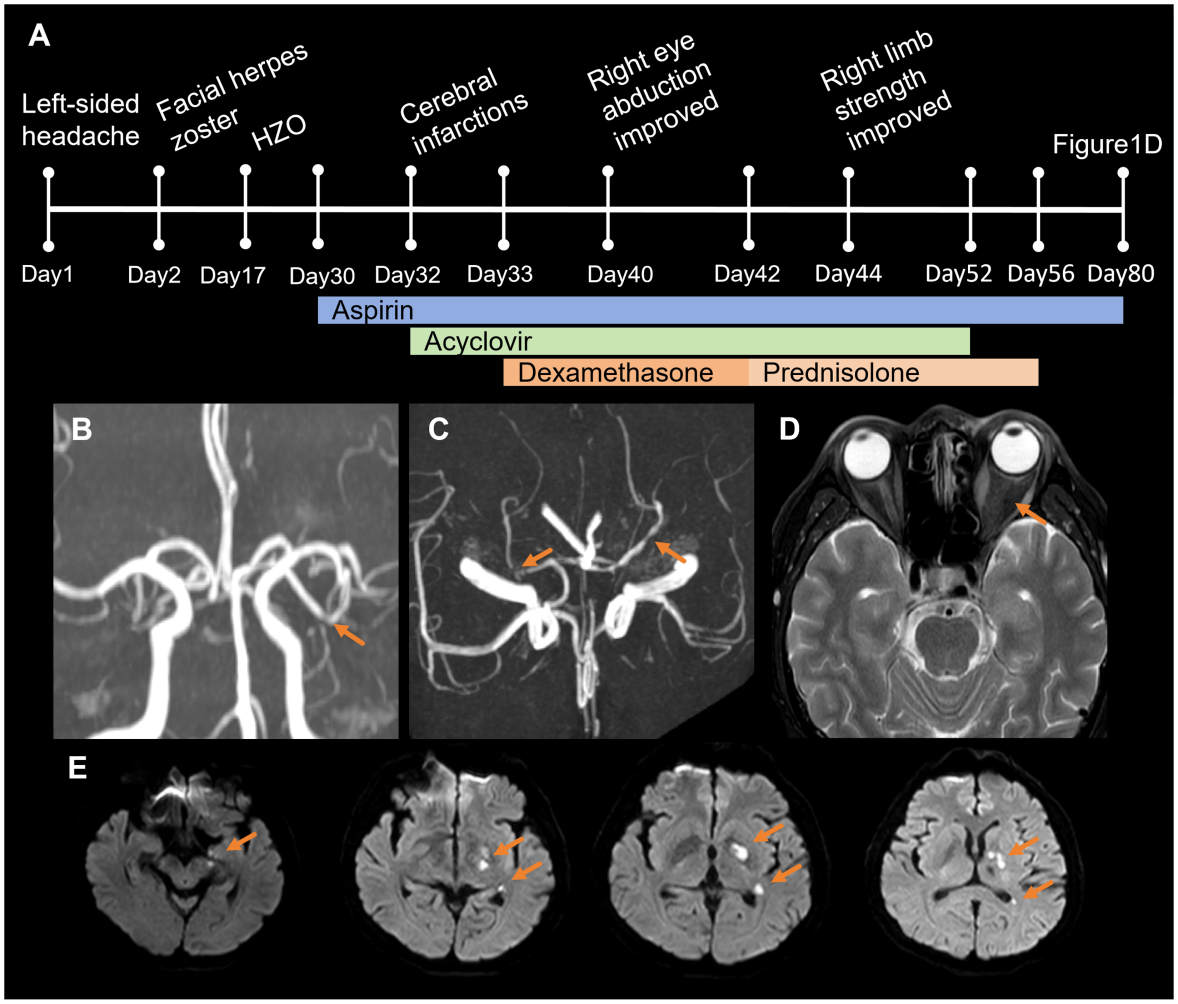
Timeline and magnetic resonance imaging. **(A)** Timeline summarizes the disease course and treatment. **(B,C)** Magnetic resonance angiography reveals that the lumen of the left middle cerebral artery is slightly narrowed in the M2 and M3 segments and limited slight stenosis in P1 segment of bilateral posterior cerebral arteries. **(D)** T2-hypointensity of the left optic nerve. **(E)** Diffusion-weighted imaging shows acute cerebral infarction in the left cerebral peduncle, parahippocampal gyrus, posterior limb of the internal capsule, globus pallidus, and paraventricular region.

A 61-year-old male initially presented with severe intermittent left-sided headache. Day 2 post-onset, he developed cutaneous left-sided facial herpes zoster, involving the root, dorsum, and tip of the nose (Hutchinson’s sign) (Figure 1A). By day 17, he developed conjunctivitis in the left eye and began experiencing diplopia. The neurological examination revealed complete ophthalmoplegia of the left eye. He was diagnosed with herpes zoster ophthalmicus (HZO) and treated with ganciclovir ophthalmic gel at an outside hospital. On day 30, the patient developed limited abduction of the right eye and right-sided hemiparesis (Figures 1B,C). The full disease course and treatment timeline are summarized in Figure 2A.

Upon referral to our institution on day 32, a detailed neurological examination was performed, which revealed anisocoria (an irregular, 4-mm left pupil vs. a regular, >2 mm right pupil), sluggish direct and consensual light reflexes in the left eye, reduced visual acuity in the left eye, complete ophthalmoplegia of the left eye, impaired abduction of the right eye, right-sided hemiparesis (muscle strength was graded V-in the right upper limb and III in the right lower limb), and positive Babinski and Chaddock signs on the right. Subsequently, cranial and ocular magnetic resonance imaging (MRI) revealed acute cerebral infarction in the left cerebral peduncle, parahippocampal gyrus, posterior limb of the internal capsule, globus pallidus, and paraventricular region (Figure 2E). A reduced T2 signal intensity was also noted in the left optic nerve (Figure 2D). Magnetic resonance angiography (MRA) revealed mild luminal narrowing in the M2 and M3 segments of the left middle cerebral artery and mild stenosis in the P1 segments of both posterior cerebral arteries (Figures 2B,C). Visual evoked potential showed prolonged latency of the left P100. The cerebrospinal fluid (CSF) analysis demonstrated a slightly elevated opening pressure of 190 mmH_2_O, along with lymphocytic pleocytosis (26 white blood cells/mm^3^, 84% lymphocytes) and the presence of VZV DNA detected by next-generation sequencing (46 reads). Furthermore, screening for autoimmune antibodies was negative (Table 1).

**Table 1:** Cerebrospinal fluid (CSF) analysis results.

CSF	Measured value	Reference
Opening pressure (mmH_2_O)	190↑	80–180
White blood cell (/mm^3^)	26↑	0–5
Lymphocytes (%)	84↑	60–70
Chloride (mmol/L)	121.5	111–130
Protein (mg/L)	426	150–450
Glucose (mmol/L)	3.74	2.8–4.5
VZV DNA sequences (NGS reads)	46 (+)	Negative
Autoimmune screens	Negative	Negative

Autoimmune screens: Anti-GQ1b IgG, Anti-GD1b IgG, Anti-GM1 IgG, Anti-AQP4 IgG, Anti-MBP IgG, Anti-MOG IgG.

Based on the above results, he was diagnosed with bilateral multiple cranial neuropathies (left optic [II], oculomotor [III], trochlear [IV], trigeminal [V], and abducens [VI] nerves consistent with orbital apex syndrome (OAS); right abducens [VI] nerve) and vasculitic cerebral infarction caused by VZV. Prior to admission, on day 30, oral aspirin 100 mg daily was initiated immediately after the onset of right-sided hemiparesis. In view of the persistently elevated risk of recurrent ischemic stroke during the first year following VZV reactivation, we recommended long-term antiplatelet therapy with aspirin for at least 1 year, if tolerated, as secondary prevention of VZV vasculopathy. Upon admission on day 32, the patient was initiated on intravenous acyclovir (10 mg/kg every 8 h) for severe HZO. Subsequent lumbar puncture confirmed VZV CNS infection, and the 21-day course of acyclovir was completed. Renal function was monitored throughout treatment; the estimated creatinine clearance remained consistently >50 mL/min/1.73 m^2^, and no dose adjustment was required. On day 33, the patient received a 9-day course of intravenous dexamethasone with a tapering regimen: 10 mg daily for 3 days, followed by 5 mg daily for 3 days, and then 2.5 mg daily for 3 days. After completion of intravenous therapy, he was transitioned to oral prednisolone at a starting dose of 15 mg daily to ensure smooth withdrawal and prevent inflammatory rebound. The oral prednisolone was tapered by 5 mg every 5 days until discontinuation (15 mg daily for 5 days, then 10 mg daily for 5 days, then 5 mg daily for 5 days, then stop). No adverse events or clinical deterioration were observed during the tapering period.

After 1 month, the patient returned for a follow-up visit, showing significant symptomatic improvement, the right eye abduction limitation had resolved, and the right limb muscle weakness had recovered substantially, while the left eye still exhibited abduction limitation (Figure 1D). The full disease course and treatment timeline are summarized in Figure 2A.

From the patient’s subjective perspective, severe headache and facial pain significantly disrupted his daily life. The development of double vision and complete left ophthalmoplegia impaired basic activities such as reading, while subsequent right-sided hemiparesis caused unsteady gait and grasp, leaving him frustrated and anxious about recovery. At one-month follow-up, he reported substantial improvement: facial pain had resolved, right eye abduction and limb strength had normalized, enabling independent walking and self-care. He remains optimistic, noting that residual mild left eye abduction limitation has minimal impact on his daily life.

## Discussion

This case presents a striking deviation from the typical unilateral pattern of VZV infection, evolving from unilateral HZO with Hutchinson’s sign to bilateral cranial neuropathies and a strategically located cerebral infarction. Hutchinson’s sign refers to the appearance of herpes zoster lesions on the skin of the nasal tip, dorsum, and root. This sign, resulting from VZV reactivation in the nasociliary branch of the trigeminal nerve, is a well-established predictor of ocular involvement (2). HZO may present with ophthalmoplegia several weeks after onset (4), most commonly involving cranial nerves III and VI, less frequently cranial nerve IV, and rarely the optic nerve. However, the emergence of contralateral abducens palsy (CN VI) alongside bilateral cerebral arterial stenosis and a multifocal AChA territory infarction suggests an extensive and aggressive pathological process, reflecting the dual neurotropic and vasculotropic nature of VZV.

VZV-associated ophthalmoplegia typically presents as one of three distinct anatomical syndromes: orbital apex syndrome (OAS), cavernous sinus syndrome (CSS), or superior orbital fissure syndrome (SOFS) (5). These syndromes are distinguished by the anatomical site of viral involvement and the resulting pattern of cranial nerve deficits: OAS is characterized by combined involvement of cranial nerves II, III, IV, V_1_ (ophthalmic division), and VI, typically presenting with complete ophthalmoplegia and vision loss. CSS classically spares the optic nerve but may involve V₂ as well, and is often accompanied by orbital venous congestion manifestations. SOFS affects only nerves traversing the superior orbital fissure (III, IV, VI, V₁), sparing the optic nerve and resulting in ophthalmoplegia without visual impairment. In our patient, the combination of left optic neuropathy, complete ophthalmoplegia, and facial herpes in the V₁ distribution—without proptosis or bruit—is most consistent with OAS.

The mechanisms of ophthalmoplegia in VZV infection are multifaceted. Proposed hypotheses include direct viral cytopathy, an immune-mediated response, occlusive vasculitis, and reactivation of other latent neurotropic viruses (6). In our patient, several observations support the possibility of direct viral involvement in the cranial neuropathies, although other mechanisms cannot be excluded. Firstly, the screening for autoimmune antibodies was negative, arguing against a robust para-infectious immune-mediated process. Secondly, no other neurotropic viruses were detected in the CSF, making viral reactivation less likely. Most critically, the pattern and progression of cranial nerve involvement argue against isolated vasculitic infarction. Although our patient’s MRI did reveal an acute ischemic lesion in the left cerebral peduncle, this structure is part of the midbrain and is anatomically distinct from the right abducens nucleus, which is located in the pons. The absence of an acute infarct in the dorsal pons argues against an isolated vasculitic event as the cause of the right abducens nerve (CN VI) palsy.

For the CN VI palsy in this patient, the most plausible etiological mechanisms are non-vasculitic, and can be attributed to one or a combination of the following: raised intracranial pressure (ICP) with a false localizing sign, mild contralateral cavernous sinus inflammation via CSF/leptomeningeal spread, or possible brainstem parenchymal spread. First, raised ICP is a key contributor to the false localizing sign of right CN VI palsy. The patient presented with a CSF opening pressure of 190 mmH_2_O, which is slightly elevated above the normal reference range. The abducens nerve is the most common false localizing cranial nerve sign in meningitis. This susceptibility arises from its anatomical features, as it has a long, free course along the clivus within the subarachnoid space, with its proximal end fixed at the pons and its distal end anchored in Dorello’s canal. Consequently, when intracranial hypertension causes downward displacement of the brainstem, the nerve is prone to bowstring-like traction between these two fixed points (7). This mechanical stretch can induce isolated CN VI palsy even in the absence of direct focal injury to the nerve or its nucleus. Second, mild inflammatory spread of VZV via the CSF/leptomeningeal route to the right cavernous sinus should be considered as another core mechanism. The abducens nerve is the only cranial nerve traversing the cavernous sinus that is not protected by the dural wall (8), a unique anatomical feature that renders it highly susceptible to selective injury from subtle, non-imaging-detectable inflammatory changes in the cavernous sinus. Even in the absence of overt radiological manifestations of cavernous sinus involvement, mild VZV-induced inflammation in this region is sufficient to cause isolated right CN VI palsy. Of note, VZV meningitis frequently presents without meningeal signs. In a series of 123 PCR-confirmed VZV meningitis cases, neck stiffness and fever were absent in a substantial proportion of patients (9). Although the patient lacked clinical meningeal signs, the presence of CSF pleocytosis and positive VZV DNA sequences confirms the diagnosis of VZV meningitis. Therefore, basilar meningitis should be considered as a plausible underlying mechanism rather than being excluded.

**Figure 3:**
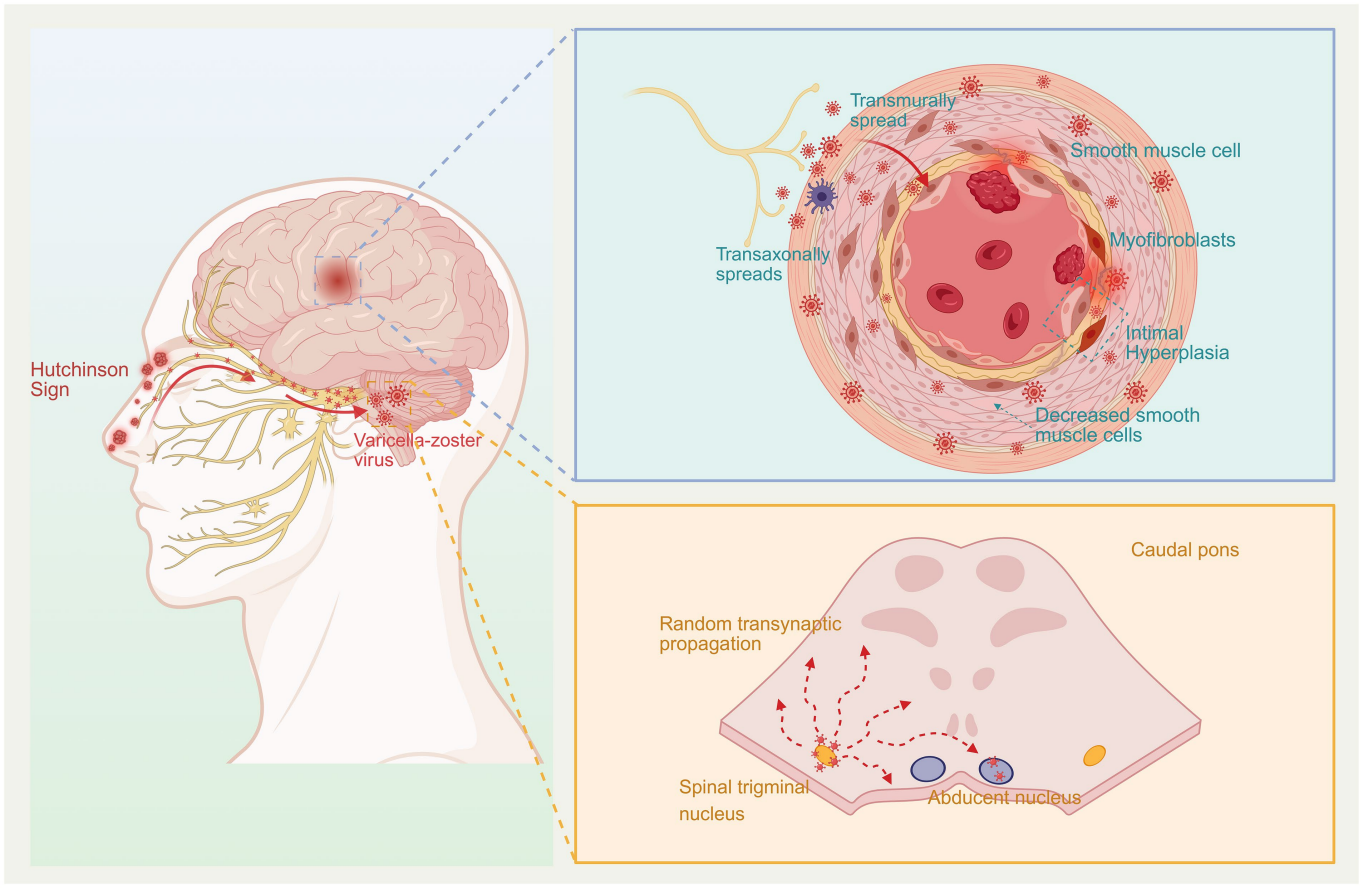
Upon reactivation, VZV induces neurological complications via a dual mechanism: (1) The virus spreads transaxonally to the arterial adventitia, followed by transmural invasion, triggering vasculitis, intimal hyperplasia, and luminal stenosis, ultimately leading to cerebral infarction. (2) Concurrently, the virus undergoes retrograde transport along the trigeminal nerve to the spinal trigeminal nucleus in the brainstem, with subsequent transsynaptic spread to the contralateral abducens nucleus, resulting in contralateral abducens nerve palsy. Created with BioRender.com.

VZV brainstem parenchymal spread is a possible mechanism for right abducens nerve palsy. According to the literature, the transaxonal spread of VZV may occur bidirectionally, and reactivated VZV can undergo retrograde axonal transport along the trigeminal nerve to the spinal trigeminal nucleus tract (10). Based on this mechanism, it can be hypothesized that VZV was retrogradely transported via the left trigeminal nerve to the ipsilateral brainstem and then potentially reached the contralateral (right) abducens nucleus via transsynaptic propagation (11) (Figure 3). The 13-day interval between the onset of HZO and the right CN VI palsy matches the reported incubation period for VZV, which ranges from 10 to 21 days (12), thereby accounting for the delayed onset of symptoms. However, given the absence of direct imaging or pathological evidence, this mechanism should be considered as one of several potential pathways. In summary, the contralateral CN VI palsy in this patient likely resulted from a combination of factors—including elevated intracranial pressure, basilar meningeal inflammation, possible cavernous sinus involvement, and direct viral invasion of the brainstem. This multifactorial etiology is consistent with the complex neurotropic patterns of VZV infection.

In patients presenting with ocular herpes zoster and unexplained cerebral infarction, the detection of VZV DNA in the cerebrospinal fluid confirms the diagnosis of VZV vasculopathy. Evidence indicates a significantly elevated risk ratio for stroke following HZO, with these patients exhibiting a mean 4.2-fold increased risk (13). The first year post-infection represents the most critical period for stroke, particularly the first 3 months (13), which is consistent with the clinical course observed in this case. Either large or small vessels can be involved: both are affected in 50% of cases, while isolated small artery involvement accounts for 37% and isolated large artery involvement for only 13% (14).

Evidence suggests that VZV spreads transaxonally to cerebral arteries via sensory nerve fibers originating from the trigeminal ganglion. This propagation triggers VZV vasculopathy, characterized by a decrease in smooth muscle cells within the arterial media and intimal hyperplasia due to the abnormal aggregation of smooth muscle cells and myofibroblasts. Collectively, these pathological changes disrupt arterial diameter and contractility, which elevates the risk of thrombosis and ultimately leads to cerebral infarction (15) (Figure 3). VZV spreads to cerebral arteries via the afferent fibers of the trigeminal ganglion. Compared to the posterior circulation vessels, the middle cerebral artery and the internal carotid artery have denser innervation from the trigeminal nerve, making them more susceptible to VZV vasculopathy (16). We speculate that VZV has reactivated within the trigeminal nerve and caused ophthalmic herpes zoster through the ophthalmic branch, eventually reaching the intracranial arteries via afferent sensory nerves. The virus then spreads transmurally from the adventitia to the intima of the arteries, inducing inflammation of the intima, destruction of the arterial wall, and consequently, leading to cerebral infarction. The involved areas in this patient, including the posterior limb of the internal capsule, paraventricular region, globus pallidus, parahippocampal gyrus, and cerebral peduncle, collectively indicate involvement within the territory of the AChA (3). The punctate infarctions observed in the cerebral peduncle and parahippocampal gyrus could easily be mistaken for posterior cerebral artery territory involvement. However, in accordance with the principle of parsimony (Occam’s razor) and the overall pattern of ischemic involvement in this patient, these findings are consistent with ischemia affecting the terminal perforating AChA following its occlusion. They are more accurately characterized as “collateral damage” resulting from a primary insult to the main trunk of the AChA, rather than representing the “epicenter” of an ischemic event attributable to the posterior circulation.

## Conclusion

Our patient exhibited both multifocal cranial neuropathies, reflecting the complex pathogenesis of VZV neuroinfection, and diffuse vasculopathy, evidenced by MRA and cerebral infarction. This co-occurrence underscores the dual neurotropic and vasculotropic nature of VZV. This case expands the known spectrum of neurological complications of HZO. It serves as a critical reminder that patients presenting with Hutchinson’s sign require not only early and aggressive antiviral therapy but also prolonged neurological vigilance, even weeks into the illness. The development of any new neurological sign, especially contralateral or bilateral symptoms, should prompt immediate investigation with CSF analysis and neuroimaging to diagnose VZV vasculopathy or encephalitis early. Future studies focusing on the molecular determinants of VZV’s neurotropic and vasculotropic pathogenesis could provide deeper insights into the pathogenesis of these severe complications.

## Data Availability

The raw data supporting the conclusions of this article will be made available by the authors, without undue reservation.
